# Neuroprotective Potential of Sodium-Glucose Cotransporter-2 (SGLT2) Inhibitors in Type 2 Diabetes: A Narrative Review

**DOI:** 10.7759/cureus.97103

**Published:** 2025-11-17

**Authors:** Rupanshu R, Ajaybir S Buttar

**Affiliations:** 1 Internal Medicine, St Martinus University Faculty of Medicine, Willemstad, CUW; 2 Internal Medicine, Shri Guru Ram Institute of Medical and Health Science, Dehradun, IND

**Keywords:** alzheimer's disease, dapagliflozin, dementia, empagliflozin, neurocognitive impairment, neuroprotection, sodium-glucose cotransporter-2 (sglt2) inhibitors, t2dm, type 2 diabetes mellitus (dm)

## Abstract

Once primarily celebrated for their glucose-lowering effect and their defense of the heart and kidneys, sodium-glucose cotransporter-2 (SGLT2) inhibitors are now at the center of a compelling new research question: do their benefits extend to the brain? As dementia rates climb globally, this can be linked with the rising prevalence of type 2 diabetes. With this, the search for neuroprotective strategies has become an urgent concern. This narrative review aims to navigate the current evidence to determine whether these drugs can protect patients with diabetes from cognitive decline. We uncover a fascinating dichotomy: a vast array of real-world observational data, encompassing hundreds of thousands of patients, consistently points toward a significant neuroprotective effect, suggesting that SGLT2 inhibitor use is associated with a markedly lower risk of dementia compared to other antidiabetic therapies such as dipeptidyl peptidase-4 (DPP-4) inhibitors or sulfonylureas.

Unfortunately, this promising signal is met with silence from the highest level of evidence available to us, namely, evidence from meta-analyses of randomized controlled trials (RCTs), which, although methodologically rigorous, find no such association. We attempted to argue that this result is not a contradiction but rather a reflection of a scientific puzzle shaped by the limitations of current research. Observational studies offer the necessary long-term view but are susceptible to bias, while existing trials were too short and ill-equipped to capture the long latency of neurodegeneration. Delving deeper, we explore the powerful biological reasoning for neuroprotection, which includes reducing neuroinflammation and improving cerebral blood flow, where SGLT2 inhibitors may even rescue the brain from an energy crisis by providing it with an alternative fuel of ketones instead of glucose.

The current landscape, therefore, is one of cautious optimism. While it is too soon to declare any kind of victory, the convergent evidence from real-world data and strong plausibility presents a powerful case for potential, demanding definitive answers from a new generation of focused, long-term clinical trials.

## Introduction and background

The number of people living with dementia is expected to triple from 50 million to 152 million by 2050, and it is a growing global public health challenge according to the WHO [1]. Dementia represents an extensive clinical syndrome characterized by progressive decline in memory, cognition, and daily functional abilities [2]. Dementia refers to a group of symptoms affecting memory, social abilities and thinking enough to interfere with daily functioning, and the ability to perform daily activities [2]. Among various types, Alzheimer’s disease is the most common type of dementia, accounting for nearly two-thirds of all cases worldwide [2]. Alzheimer's disease is characterized by A-beta (Aβ) amyloid accumulation in the brain and neuronal loss, resulting in impaired memory, behavior, and ultimately, loss of activities of daily living [2].

Since their FDA approval in 2013, sodium-glucose cotransporter-2 inhibitors (SGLT2) have been primarily recognized as glucose-lowering agents for patients with type 2 diabetes mellitus (T2DM) by promoting glucose excretion through the urine [3]. Common examples include empagliflozin, dapagliflozin, and canagliflozin [3]. Their cardiovascular effects, particularly in reducing heart failure with both preserved and reduced ejection fraction, are associated with lower hospitalization rates and mortality in these patients [3]. It was clearly established that SGLT2 inhibitors were not just a therapy for glucose control, but their effects are far broader through several trials and meta-analyses.

Recent preclinical and research studies have also demonstrated their effects in improving cognition. They improve neuroinflammation, enhance the brain’s metabolic efficiency, and may have a neuroprotective effect [4]. These properties clearly raise the possibility that they could help lower the risk of dementia or even slow other neurodegenerative disorders [4]. While the evidence remains mixed and limited by study design, some observational cohort studies have suggested a significant reduction in rates of cognitive decline among patients taking SGLT2 inhibitors [4].

This growing field of work reflects a paradigm shift in how we view SGLT2 inhibitors, from primarily being used as cardioprotective and glucose control agents to those that may also impact nearly every major organ system, including the brain. Understanding their neurocognitive effects could open new frontiers not just in diabetes care, but in the prevention and management of conditions such as Alzheimer’s disease and other neurocognitive disorders.

**Figure 1:**
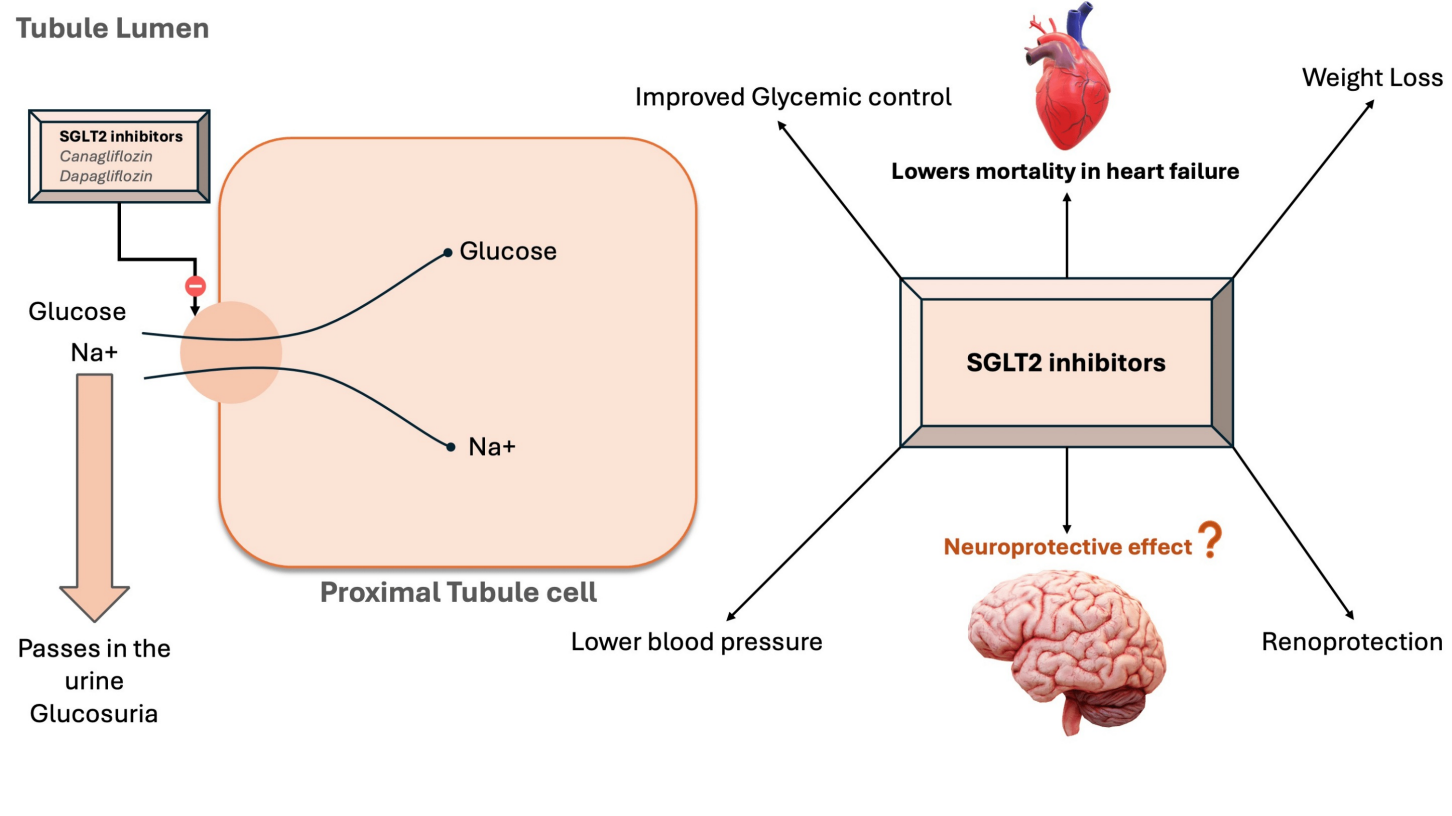
Mechanism of action and known effects of SGLT2 inhibitors SGLT2: Sodium-glucose cotransporter-2; Na+: sodium Image credits: Rupanshu

Our narrative review tries to answer the following question: In patients with T2DM, is treatment with SGLT2 inhibitors effective in reducing the risk of cognitive decline, dementia, or even improving neurological outcomes? Figure *1* shows the known effects of SGLT-2 inhibitors and their mechanism of action.

## Review

Methods

The selection process for our narrative review included studies based on a set of eligibility criteria. Only the articles written and published in English were included. Articles published within the last five years, focusing on human subjects, were taken into consideration. The research focus was also on the effects of SGLT2 inhibitors across all age groups. Table 1* *summarizes our search strategies, databases used, and the number of articles identified from each strategy as of October 12, 2025. 

**Table 1:** Summary of search strategies MeSH: Medical Subject Headings

Search strategy	Database used	Number of articles identified
((("Diabetes Mellitus, Type 2/therapy"[Mesh]) AND "Sodium-Glucose Transporter 2 Inhibitors/therapeutic use"[Mesh]) AND "Cognitive Dysfunction"[Mesh])	PubMed (MeSH)	10
(((type 2 diabetes mellitus) OR (T2DM)) AND ("SGLT2 inhibitor" OR "empagliflozin" OR "dapagliflozin" OR "canagliflozin")) AND ("cognitive decline" OR "dementia" OR "cognitive impairment" OR "Alzheimer disease")	PubMed	33
("type 2 diabetes mellitus"[Title/Abstract]) AND (empagliflozin[Title/Abstract] OR dapagliflozin[Title/Abstract] OR canagliflozin[Title/Abstract]) AND ("cognitive impairment"[Title/Abstract] OR "Alzheimer disease"[Title/Abstract] OR dementia[Title/Abstract])	PubMed Central	7
(((type 2 diabetes mellitus) OR (T2DM)) AND ("SGLT2 inhibitor" OR "empagliflozin" OR "dapagliflozin" OR "canagliflozin")) AND ("cognitive decline" OR "dementia" OR "cognitive impairment"OR "Alzheimer disease")	Clinical Trials	14
("type 2 diabetes mellitus") AND (SGLT2 OR "sodium-glucose cotransporter 2" OR gliflozin) AND ("cognitive decline" OR "cognitive impairment" OR cognition OR dementia)	Science Direct	15
("type 2 diabetes mellitus" OR T2DM) AND ("SGLT2 inhibitor" OR empagliflozin OR dapagliflozin OR canagliflozin) AND ("cognitive decline" OR dementia OR "cognitive impairment" OR "Alzheimer disease")	Cochrane Library	2

Discussion

The relationship between T2DM and dementia is well-established, with shared pathophysiological pathways that include insulin resistance, chronic inflammation, oxidative stress, as well as vascular dysfunction, creating a high-risk environment for neurodegeneration [4]. The advent of SGLT2 inhibitors, a class of medications with various effects extending beyond glycemic control, such as cardiovascular and renal protection, has raised a vital question: do these benefits extend to the brain? This review synthesizes the current, often conflicting, evidence from observational studies, randomized controlled trials (RCTs), and meta-analyses to evaluate the potential of SGLT2 inhibitors in reducing the risk of cognitive decline, dementia, and other related neurodegenerative disorders in patients with T2DM. 

Evidence From Observational Studies: A Consistent Pattern of Neuroprotection

A huge body of real-world evidence, derived from extensive, population-based retrospective cohort studies, consistently suggests an association between the use of SGLT2 inhibitors and a significantly reduced risk of incident dementia in patients with T2DM. These studies, leveraging clinical databases from diverse populations, provide a strong pattern of potential neuroprotection.

A common and methodologically robust approach to make the studies more statistically as well as clinically significant is to use a comparison group, particularly dipeptidyl peptidase-4 (DPP-4) inhibitors. This design helps to mitigate confounding, as both drug classes are often prescribed as second or third-line therapies in T2DM patients. Several extensive studies employing this comparison have reported significant benefits of SGLT2 inhibitors. A German retrospective cohort study by Sarabhai et al., which included 38140 adult patients with T2DM, found that SGLT2 inhibitor use was associated with a 20% relative risk reduction for dementia compared to DPP-4 inhibitors (hazard ratio (HR): 0.80; 95% confidence interval (CI): 0.70-0.93) [4]. This protective effect was noted to be even more pronounced in male patients and in the very elderly (age >80 years) with HR: 0.77 and HR: 0.75, respectively [4]. 

An even more striking effect was observed in a study by Chiang et al., in which SGLT2 inhibitor use was associated with lower risks of overall dementia (HR: 0.66; CI: 0.59-0.74), degenerative dementia (HR: 0.68; CI: 0.60-0.76), and vascular dementia (HR: 0.59, CI: 0.49-0.70) compared to DPP-4 inhibitor use in the same propensity-matched cohort [5]. A population-based study of over 106903 individuals by Wu et al. also inferred that SGLT2 inhibitors, compared with DPP-4 inhibitors, were associated with a lower risk of dementia (adjusted hazard ratio (aHR): 0.80; CI: 0.71-0.89) over a mean follow-up of 2.80 years [6]. This study took a step forward by attempting to answer the question of which among the different SGLT2 inhibitors currently approved by the FDA imparts the highest benefit in neurocognitive behaviors. When stratified among different SGLT2 inhibitors, dapagliflozin exhibited the lowest risk (aHR: 0.67; CI: 0.53-0.84), followed by empagliflozin (aHR: 0.78; CI: 0.69-0.89), whereas canagliflozin showed no significant association (aHR: 0.96; CI: 0.80-1.16) [6]. It pointed out that among the SGLT2 inhibitors, not all conferred the same benefit, and canagliflozin showed no benefit [6].

Similarly, a large retrospective cohort analysis by Liu et al. using the TriNetX database, which included over 160000 matched patients aged ≥65 with T2DM, found that SGLT2 inhibitor use was associated with a significantly lower risk of new-onset dementia compared to DPP-4 inhibitors (HR: 0.54; 95% CI: 0.51-0.57) [7]. This protective effect extended to both Alzheimer's disease (HR: 0.53; 95% CI: 0.48-0.60; p<0.001) and vascular dementia (HR: 0.52; 95% CI: 0.46-0.58; p<0.001) [7].

A population-based cohort study from the UK by Wang et al. also compared new users of SGLT2 inhibitors against DPP-4 inhibitors and found a potential, though not statistically significant, reduction in the risk of all-cause dementia (adjusted HR: 0.77; 95% CI: 0.57-1.05) and a more specific reduced risk of vascular dementia [8]. 

Comparisons against other classes of antidiabetic agents have also shown similar conclusions. In a study by Tang et al., comparing new users of SGLT2 inhibitors with those initiating sulfonylureas, a significantly lower risk of all-cause dementia was reported (risk difference: -2.5%; 95% CI: -3.0% to -2.1%) [9]. It included about 35,000 individuals with T2DM, of whom only 1.8% in the SGLT2 inhibitor group developed all-cause dementia, compared to 4.7% in the sulfonylurea group [9]. A nationwide longitudinal cohort study from Taiwan by Siao et al. compared over 103000 SGLT2 inhibitor users to an equal number of propensity score-matched non-users [10]. This analysis revealed a statistically significant 11% lower risk of incident dementia associated with SGLT2 inhibitor use (adjusted HR: 0.89; 95% CI: 0.82-0.96) [10]. A study by Hong et al. in Hong Kong was conducted to compare SGLT2 inhibitors with glucagon-like peptide-1 receptor agonists (GLP-1 RA) and included 12489 patients initiating SGLT2 inhibitor treatment and 1075 patients initiating dulaglutide treatment [11]. It also continued the trend observed in the above observational studies, with an estimated risk ratio (RR) of 0.81 (CI: 0.56-1.16) [11]. Although according to the authors, residual confounding may be present, this study did not adjust for different levels of HbA1c or duration of diabetes. Moreover, it only considered older GLP-1 RAs, such as dulaglutide, but not newer agents like semaglutide (Ozempic) or tirzepatide (Mounjaro/Zepbound) [11].

Expanding the evidence base to major first-line agent metformin, a large retrospective cohort study by Sun et al. provided a head-to-head comparison of SGLT2 inhibitors against metformin [12]. Among nearly 75000 matched pairs, SGLT2 inhibitor use was associated with a significantly lower incidence of overall dementia compared to metformin (2.7% vs. 6.9%; adjusted HR: 0.80; 95% CI: 0.76-0.84) [12]. The risk reduction was observed for both Alzheimer's and vascular dementia subtypes and was particularly more pronounced in patients aged ≥80 years [12].

The variability in the magnitude of the observed protective effect across these studies warrants careful consideration. The reported risk reductions range from a modest 11% to a dramatic 41%. This variation appears to be heavily influenced by the choice of the comparator group. The largest risk reductions are often reported in studies comparing SGLT2 inhibitors to older drug classes, such as sulfonylureas, or to non-user T2DM patients. In contrast, comparisons against a more modern, metabolically neutral class like DPP-4 inhibitors tend to yield a more modest decrease of 20%, though still significant as shown by Wu et al. [6]. This pattern suggests the potential influence of residual confounding, particularly the "healthy user" effect. Patients prescribed newer, more expensive medications like SGLT2 inhibitors may be systematically different from those prescribed older agents; they might be more involved with their healthcare, have better socioeconomic status, or have fewer comorbidities that would prevent the use of a newer agent.

Furthermore, comparing SGLT2 inhibitors with sulfonylureas, a class notorious for causing hypoglycemia, which is itself a risk factor for cognitive decline, may artificially inflate the apparent benefit of the SGLT2 inhibitor. Therefore, the active comparator design using DPP-4 inhibitors likely provides a more realistic estimate of the actual effect size, suggesting a potential risk reduction in the range of 10%-20%. While this can limit the enthusiasm generated by some of the larger risk reductions of 30%-40%, it reinforces the consistency of SGLT2 inhibitors in the protective effect observed in real-world settings. It highlights the critical need for validation through RCTs. It also highlights the importance of studies that compare SGLT2 inhibitors to newer GLP-1 agonists like semaglutide, which have also been gaining popularity in recent years. The following Table 2 summarizes the observational studies we discussed above and their respective outcomes.

**Table 2:** Summary of observational studies and their outcomes DPP-4: dipeptidyl peptidase-4; GLP-1 RA: glucagon-like peptide-1 receptor agonists; SGLT2i: sodium-glucose cotransporter-2; HR: hazard ratio; aHR: adjusted hazard ratio; RR: risk ratio

Author (year)	Study design	Population (N)	Comparator	Mean follow-up	Primary cognitive outcome (HR/OR (95% CI))
Sarabhai et al. [4]	Retrospective cohort	38140	DPP-4 inhibitors	Maximum 5 years	Dementia: HR 0.80 (0.70-0.93) p-value 0.002
Chiang et al. [5]	Retrospective cohort	Each group consisted of 15901 patients	DPP-4 inhibitors	Mean of 2.52 years	Dementia: HR 0.66 (0.59-0.74)
Wu et al. [6]	Retrospective cohort	106903	DPP-4 inhibitors	Mean of 2.80 years	aHR 0.80 (0.71-0.89)
Liu et al. [7]	Retrospective cohort	160752	DPP-4 inhibitors	Not specified	Dementia: HR 0.54 (0.51-0.57)
Wang et al. [8]	Retrospective cohort	33008	DPP-4 inhibitors	1.3 years (median)	Dementia: aHR 0.77 (0.57-1.05)
Tang et al. [9]	Retrospective cohort	35458	Sulfonylureas	3.2 years	All-cause dementia: risk difference -2.5% (-3.0% to -2.1%)
Siao et al. [10]	Retrospective cohort	206494	Non-SGLT2i users	Not specified	Dementia: aHR 0.89 (0.82-0.96) p-value 0.0021
Hong et al. [11]	Retrospective cohort	13564	Dulaglutide (GLP-1 RA)	4.4 years (median)	Dementia: RR 0.81 (0.56-1.16)
Sun et al. [12]	Retrospective cohort	149950	Metformin	Not specified	Dementia: aHR 0.80 (0.76-0.84)

Evidence From Randomized Controlled Trials: A Conspicuous Lack of Association

In stark contrast to the consistent findings from the vast observational data, the highest level of clinical evidence, which is derived from meta-analyses of large-scale RCTs, has thus far failed to demonstrate a significant association between SGLT2 inhibitor treatment and a reduced risk of dementia. These trials, primarily designed as cardiovascular outcome trials (CVOTs) to meet regulatory requirements for cardiovascular safety, represent the most rigorous test we can do of a drug's efficacy, minimizing the biases that can be inadvertently present in observational research.

A meta-analysis by Jaiswal et al., consisting of data from 12 RCTs that included a total of 74442 patients [3]. The analysis found no significant association between SGLT2 inhibitor use and the risk of all-cause dementia (odds ratio (OR): 1.37; 95% CI: 0.70-2.69), Alzheimer's (OR 1.99; 95% CI, 0.59-6.71), or even vascular dementia (OR 0.40; 95% CI, 0.09-1.85) when compared with control groups, which were predominantly placebo [3]. The CIs for these estimates are wide, and all had the null value of 1.0 in their CI, indicating a lack of a statistically significant effect [3]. It is also noteworthy that the point estimate for all-cause dementia was above 1.0 (OR 1.37), which might suggest a trend toward harm, though this was not statistically significant [3]. 

This null finding is supported by other systematic reviews as well. Xu et al. conducted a comprehensive meta-analysis of 52 trials/publications, encompassing 111376 participants, and similarly reported no significant effect of SGLT2 inhibitors on the incidence of dementia (RR: 1.29; 95% CI: 0.78-2.12) [13]. Of note, in this meta-analysis, SGLT2 inhibitors showed slight effects to reduce the risk of Parkinson's disease, especially in major heart failure groups [13]. There was also concern of syncope and carotid artery occlusion with the use of empagliflozin and dapagliflozin [13]. It was shown that there is an increased risk of syncope (RR:1.65; 95% CI: 1.15-2.38; P<0.01) and carotid artery occlusion (RR: 1.65; 95% CI: 1.04-2.61; P=0.03) with the use of these medications, which can also be worth paying attention to side effects [13]. These side effects should be taken into account, given that syncope and carotid artery occlusion leading to stroke can be fatal, particularly in the elderly patient population, in which falls are a major cause of morbidities and mortality.

Furthermore, a systematic review and meta-analysis by Seminer et al., which evaluated 26 RCTs of various cardioprotective glucose-lowering agents, including 12 trials of SGLT2 inhibitors in one group, 10 trials of GLP-1RA, and one trial of pioglitazone as well [2]. Notably, no trials of the most common anti-diabetic medication, metformin, were identified in this meta-analysis [2]. It concluded that SGLT2 inhibitors were not associated with a reduction in cognitive impairment or dementia (OR: 1.20; 95% CI: 0.67-2.17). This review is particularly insightful as it found that another modern class of antidiabetic drugs, GLP-1 RA, was associated with a statistically significant reduction in dementia risk, suggesting that the statistically insignificant finding for SGLT2 inhibitors was not simply a feature of all modern antidiabetic therapies in these trials [2]. GLP-1RA showed promising results for reducing dementia (OR: 0.55; 95% CI: 0.35-0.86) [2]. This dichotomy of different evidence types is of value to further fuel studies, underscoring the need for further RCTs and meta-analysis. Table 3 summarizes the RCT we discussed in this section.

**Table 3:** Summary of RCTs discussed RCT: randomized controlled trials; OR: odds ratio; RR: relative risk; SGLT2i: sodium-glucose cotransporter-2 inhibitors

Author (year)	Number of RCTs included	Total patient population (N)	Comparator	Mean follow-up	Pooled effect size for dementia (OR/RR (95% CI))
Seminer et al. [2]	12 (SGLT2i trials) but a total of 26 RCTs	Not specified for SGLT2i subset; Total 164531	Placebo/control	Not specified	OR 1.20 (0.67-2.17)
Jaiswal et al. [3]	12	74442	Placebo/control	Mean of 2.9 years	OR 1.37 (0.70-2.69)
Xu et al. [13]	52	111376	Placebo	≥24 weeks	RR 1.29 (0.78-2.12)

Reconciling the Dichotomy of Different Evidence Types: Methodological Limitations and the Nature of Dementia

The stark discrepancy between the findings of observational studies and RCTs presents a critical scientific puzzle. However, this discrepancy may not represent a direct contradiction but rather a portrayal of the distinct questions each study design is capable of answering and the inherent methodological constraints each faces when applied to a long-latency disease like any dementia, particularly Alzheimer’s.

The RCTs included in the meta-analyses, while methodologically rigorous, were not designed to answer the question of dementia prevention. Their primary limitations in this context are threefold. First is the issue of the endpoint itself. These were CVOTs, designed to assess major adverse cardiovascular events (MACE) over a few years. Cognitive outcomes were typically not pre-specified endpoints and were often collected passively as adverse events reported by investigators or patients. This method is prone to significant selection bias and misclassification of dementia cases, lacking the systematic, protocol-driven cognitive assessments necessary for a reliable outcome measure. Second, and perhaps most critically, is the timeline. The pathophysiology of dementia unfolds over decades. The mean follow-up duration in the Jaiswal et al. meta-analysis was a mere 2.9 years, or just greater than 24 weeks for the meta-analysis by Xu et al. [3,13]. This timeframe is almost certainly insufficient to observe a meaningful difference in the incidence of a slowly progressing neurodegenerative disease. A preventative therapy would need to be administered for many years, likely over a decade or more, before its effect on dementia incidence would become apparent. Third, the patient populations in CVOTs are selected for high cardiovascular risk, which may not be fully representative of the broader T2DM population at risk for dementia through other, non-vascular pathways, such as primary amyloid or hyperphosphorylation of tau pathology. These patients with already high cardiovascular risk represent a portion of the population who are already at a higher risk for dementia.

Conversely, while observational studies offer the advantage of long-term follow-up in large, real-world populations, they are susceptible to inherent biases that can never be fully eliminated through statistical adjustment. As previously discussed, confounding by indication and the "healthy user" effect are significant concerns. Even with sophisticated methods like propensity score matching, unmeasured variables (e.g., health-seeking behaviors, diet, exercise, and medication adherence) can inadvertently skew the results and lead to an overestimation of the treatment effect. 

Synthesizing these limitations leads to a more nuanced interpretation. The large observational studies, despite their potential for bias, provide a consistent signal of a potential real-world association over the long periods that are necessary for dementia to develop. They generate a very compelling hypothesis. The RCTs, on the other hand, provide a clean, unbiased assessment, but through a methodological keyhole that is too narrow in time and improperly focused on its outcomes to definitively confirm or discard this hypothesis. Therefore, the current evidence base is ultimately insufficient. The consistent signal from observational data provides a strong rationale to move forward, while the null findings from existing RCTs serve as a crucial note of caution, preventing a premature conclusion of benefit based on observational data alone. The RCTs even point to these drugs causing more harm, with syncope and carotid artery occlusion being some manifestations that give thoughtful attention to [13]. This state of evidence points unequivocally to the need for dedicated, long-term RCTs with dementia as a primary, pre-specified endpoint in a population that is much more representative of the actual T2DM population.

Impact on Intermediate Cognitive Markers and Biomarkers

While the evidence on the hard clinical outcome of dementia remains conflicting, several studies have investigated the impact of SGLT2 inhibitors on more immediate measures of cognitive function and underlying pathophysiology. These studies can provide valuable, albeit preliminary, insights into whether these agents can modify the trajectory of cognitive decline.

A prospective study by Zhang et al. offers some of the most compelling evidence in this domain [14]. The study followed patients with T2DM and pre-existing cognitive impairment who were treated with the SGLT2 inhibitor henagliflozin [14]. Over a six-month period, the henagliflozin group demonstrated a statistically and clinically significant improvement in cognitive function, as measured by the Montreal Cognitive Assessment (MoCA) [14]. The median MoCA score in this group increased from 21 to 24 (p<0.0001), whereas no significant change was observed in the non-SGLT2 inhibitor group [14]. This finding is significant as it suggests a potential for SGLT2 inhibitors to not only prevent future decline but also to improve existing cognitive impairment. 

Even more critically, the study by Zhang et al. provided a direct biochemical link to a core mechanism of neurodegeneration. Henagliflozin treatment was found to be independently associated with a significant decrease in plasma levels of phosphorylated tau181 (p-tau181), a key biomarker for the neurofibrillary tangle pathology of Alzheimer's disease [14]. At six-month follow-up, plasma p-tau181 levels were significantly decreased in all patients in the henagliflozin group (OR: 11.5, 95% CI: 10.3-13.0 vs. 9.2, 95% CI: 7.1-10.7; P<0.0001). This pivotal finding suggests that the drug may be modifying the underlying disease process, beyond what is captured by MoCA scores [14]. The key limitations of this study are twofold: the first is the short follow-up period of just six months, which may have limited the ability to detect substantial changes in MoCA scores and p-tau181 levels. Second, only henagliflozin was employed in this study, there being no comparison to other more popular SGLT2 inhibitors like dapagliflozin or empagliflozin. Although this study indicates a favorable effect of henagliflozin on reducing cognitive impairment, it also underscores the need for further investigations to determine the potential clinical benefits of different SGLT2 inhibitors in improving cognitive outcomes [14].

The evidence is not uniformly positive in all the studies here as well. A 16-week, head-to-head randomized trial by Cheng et al. compared the SGLT2 inhibitor dapagliflozin against the GLP-1 RA liraglutide and acarbose [15]. In this short-term study, dapagliflozin did not significantly improve cognitive domains, whereas liraglutide did. In this study, techniques such as functional neuroimaging, like functional magnetic resonance imaging (fMRI), are now the most important noninvasive methods for diagnosing neural activation abnormalities that occur even before pathological changes [15]. Also, it pointed out that disruption of the olfactory neural circuits has been consistently observed in patients with mild cognitive impairment and Alzheimer’s disease, and it has been confirmed as an early and sensitive indicator of cognitive decline in patients with type 2 diabetes [15]. These newer methods, along with MoCA, support the conclusion drawn from this study; while the short duration of this trial limits firm conclusions, it raises the possibility of differences in neuroprotective efficacy between antidiabetic drug classes. One other major limitation of this study was that only 55 patients were screened, and out of them, only 36 were enrolled and assigned to different groups (12 for each of the three groups) [15].

Further supporting a benefit, a case-control study by Majid et al. compared T2DM patients on SGLT2 inhibitors to those on sulfonylureas [16]. The SGLT2 inhibitor group had significantly better MoCA scores (mean 22.5 vs. 20.0) and lower PHQ-9 scores as well (8.2 vs. 6.5). The authors linked this clinical benefit to a better modulation of key neuroinflammatory biomarkers by SGLT2 inhibitors [16]. Together, these studies suggest that SGLT2 inhibitors can positively impact cognitive function and related biomarkers, though the effects may be drug-specific and require longer-term investigation to be fully understood. 

Nodirahon et al. also evaluated the dual impact of SGLT2 inhibitors on cognitive impairment and depression in T2DM [17]. Their findings revealed a drastic shift in both domains that we discussed. In their study, SGLT2 inhibitors showed a higher prevalence of mild to moderate depression (p<0.001; OR: 1.74) and cognitive impairment (p=0.039, OR: 1.32) compared to controls [17]. Rather than concluding uniformly in favor of SGLT2 inhibitors, the discussion points out the dichotomy of outcomes and the need for more high-quality RCTs specifically focused on cognitive and psychiatric endpoints [17].

Plausible Biological Mechanisms for Neuroprotection

The potential neuroprotective effects of SGLT2 inhibitors are unlikely to stem from a single mode of action. Rather, they are thought to be multifactorial, involving a mixture of metabolic, vascular, and anti-inflammatory pathways that extend well beyond their primary glucose-lowering effect. In a preclinical and clinical data analysis by Tran et al. SGLT2 inhibitors showed promising results due to their ability to improve mitochondrial function, reduce oxidative stress, and modulate neuroinflammation [18]. Although the current evidence remains preliminary, this plausibility strengthens the rationale and prompts further investigation. Listed below are the plausible mechanisms currently proposed to understand the neuroprotective effects of SGLT2 inhibitors.

Attenuation of neuroinflammation and oxidative stress: T2DM is characterized by a state of chronic, low-grade systemic inflammation, which can damage the blood-brain barrier and promote neuroinflammation, a key driver of neuronal damage in neurodegenerative diseases such as Alzheimer’s. SGLT2 inhibitors have demonstrated potent anti-inflammatory properties. Preclinical evidence suggests they may directly modulate neuroinflammation by inhibiting the NOD-like receptor protein 3 (NLRP3) inflammasome within microglia, a critical pathway implicated in the pathogenesis of Alzheimer's disease [19]. This is supported by clinical data from Majid et al., who showed that T2DM patients treated with SGLT2 inhibitors had more favorable profiles of neuroinflammatory biomarkers, including high mobility group box 1 (HMGB1), tumor necrosis factor-alpha (TNF-α), IL-1 beta, and IL-6, compared to patients treated with sulfonylureas [16]. These inhibitors limit the biomarkers of IL-18 and IL-1β by modulating the NLRP3 inflammasome in microglia [19].

Improved cerebral vascular health: Vascular dysfunction is a critical link between T2DM and cognitive decline, contributing significantly to the risk of both vascular dementia and Alzheimer's disease. SGLT2 inhibitors exert multiple beneficial effects on the vascular system. They are known to lower blood pressure, reduce arterial stiffness, and improve endothelial function. These systemic vascular improvements are likely to translate into better cerebral perfusion, enhanced integrity of the blood-brain barrier, and protection of the neurovascular unit (comprising pericytes, astrocytes, and microglia). The consistent finding in observational studies of a reduced risk of vascular dementia associated with SGLT2 inhibitor use provides strong clinical support for this vascular-mediated mechanism of neuroprotection, as shown in the study by Chiang et al., in which the risk of vascular dementia was significantly reduced (HR: 0.59; 95% CI: 0.49-0.70) compared to DPP-4 inhibitors [5].

The ketone hypothesis in modulating cerebral energy metabolism: Perhaps the most unique and compelling proposed mechanism for SGLT2 inhibitor-mediated neuroprotection relates to their ability to fundamentally alter cerebral energy metabolism. The brains of patients with Alzheimer's disease and other dementias exhibit a well-documented state of region-specific glucose hypometabolism. This creates an energy crisis where neurons are effectively starved of energy, even in the presence of normal or high peripheral glucose levels [20]. SGLT2 inhibitors address this problem through a distinct pathway. By inducing persistent glycosuria, they create a mild but sustained negative energy balance. The body responds to this by increasing hepatic ketogenesis, leading to a modest elevation in circulating levels of ketone bodies, such as β-hydroxybutyrate (BHB). Ketone bodies can readily cross the blood-brain barrier and are efficiently utilized by neurons as an alternative fuel source, effectively bypassing the defective glucose metabolic pathways that characterize the neurodegenerative brain [20]. This metabolic shift from glucose to ketones could, in fact, rescue neurons from the energy deficit, therefore improving function and promoting survival [20]. This mechanism is distinct from just simple glycemic control and could explain why SGLT2 inhibitors might offer neuroprotection independent of their effect on HbA1c. It provides a strong, testable hypothesis that distinguishes SGLT2 inhibitors from many other antidiabetic agents. One caveat when following this pathway is that SGLT2 inhibitors increase the risk of euglycemic ketoacidosis, especially in patients with low BMI or depleted glycogen reserves [21]. This side effect is one of the reasons why SGLT2 inhibitors are not approved for use in patients with type-1 diabetes [21].

Direct neuronal and synaptic effects: Emerging preclinical evidence suggests that SGLT2 inhibitors may also exert direct effects on neuronal health. While initially thought to be confined to the kidney, SGLT1 and SGLT2 receptors have now been identified in various regions of the brain, including the hippocampus, suggesting a potential for direct drug action on neuronal glucose handling and function, although this remains an area of active investigation [22]. Table 4 summarizes the different biological pathways for neuroprotection.

**Table 4:** Summary of biological pathways involved in neuroprotection TNF: tumor necrosis factor; IL: interleukin; NLRP3: nucleotide-binding oligomerization domain-like receptor protein 3

Mechanism category	Description of the mechanism	
Metabolic	Induction of mild, sustained ketosis provides an alternative fuel source (ketone bodies) for the brain, bypassing impaired glucose metabolism characteristic of neurodegeneration	
Anti-inflammatory	Reduction of systemic and central neuroinflammation through modulation of pro-inflammatory cytokines (TNF-alpha, IL-1 beta, IL-6, and IL-18) and inhibition of microglial pathways like the NLRP3 inflammasome	
Vascular	Improvement of endothelial function, reduction of blood pressure, and enhanced blood-brain barrier integrity, leading to better cerebral perfusion and reduced risk of vascular-related brain injury	
Antioxidant	Attenuation of cerebral oxidative stress and reduction of oxidative DNA damage, protecting neurons from free radical-induced injury	

Heterogeneity of Treatment Effects and Future Directions

The available evidence suggests that the potential neuroprotective benefits of SGLT2 inhibitors may not be uniform, varying both by the specific drug within the class and by the characteristics of the patient population being treated. Wu et al. observed in their cohort that while dapagliflozin and empagliflozin were associated with a lower risk of dementia, canagliflozin showed no such association, pointing to potential differences within the drug class that merit further investigation [6]. 

Furthermore, the protective association appears to be concentrated in specific patient subgroups. The benefits seem more pronounced in the very elderly (patients over 80 years) and in males, as reported by Sarabhai et al. [4]. A recurring theme that was observed is the enhanced benefit in patients with a high burden of vascular disease. Tang et al. found that patients with pre-existing cardiovascular and cerebrovascular disease derived the greatest benefit from SGLT2 inhibitors in terms of dementia risk reduction [9]. This suggests that the potent vascular effects of these drugs may be a potential driver of the observed cognitive benefits, particularly in preventing vascular dementia. It also pointed out that new users of SGLT2 inhibitors were significantly associated with a lower risk of all-cause dementia as compared to those of sulfonylureas [9]. The association also varied among different subgroups, such as Hispanic and patients with pre-existing chronic kidney disease [9].

The profound discrepancy between the consistent results in observational studies and the null findings in existing RCTs creates a clear and urgent mandate for future research, particularly RCTs that provide the highest level of scientific evidence either in favor or against SGLT2 inhibitor effects. Furthermore, it is important to note that observational studies are inherently prone to various biases that may limit the reliability of their findings, including confounding by indication and channeling bias, as patients prescribed SGLT2 inhibitors may, in fact, differ characteristically from control groups, particularly in terms of renal function and baseline health status. Our scientific community cannot just rely on potentially confounded real-world evidence, nor can it dismiss this signal based on RCTs that were not fit for the purpose of assessing a long-term neurological outcome. The definitive next step is the design and execution of large-scale, long-duration (at least five-10 years) RCTs that are specifically designed with incident dementia or a composite of significant cognitive decline as a pre-specified primary endpoint. To provide conclusive evidence and explain the underlying mechanisms, these future trials must incorporate systematic, longitudinal cognitive testing using comprehensive neuropsychological techniques and integrate advanced biomarker analysis, including plasma or CSF measures of p-tau, TNF-α, IL-1 beta, IL-6 and IL-18, as well as advanced neuroimaging techniques like fMRI scans for diagnosing neural activation abnormalities even before exact pathologies presents clinically [15].

## Conclusions

The current body of evidence regarding the role of SGLT2 inhibitors in improving cognitive decline in patients with T2DM presents a conflicting but ultimately promising picture. If we can solve this puzzle, we may have in our hands a promising treatment for preventing or slowing neurocognitive decline and dementia. However, we have discussed that, on one hand, there is a large and growing body of well-conducted observational studies that consistently report a significant association between SGLT2 inhibitor use and a reduced risk of all-cause dementia when compared with other antidiabetic agents such as sulfonylureas, metformin, GLP-1 RAs, and DPP-4 inhibitors. This signal is robust across diverse populations and healthcare systems, providing compelling real-world data that a neuroprotective effect may, in fact, exist. On the other hand, this finding is not supported by meta-analyses of existing RCTs, which, representing a higher level of evidence, show a null effect. This dichotomy is likely attributable to significant methodological limitations inherent in both study types for answering this specific question. The observational research is susceptible to residual confounding that may overestimate the true effect, while the existing RCTs are constrained by inadequate follow-up durations and a lack of focus on the primary endpoint of cognitive outcomes, rendering them ill-suited to detect a preventive effect on a long-term disease like dementia.

A strong and multifaceted biological rationale for neuroprotection can exist, centered on the unique ability of SGLT2 inhibitors to improve cerebral vascular health, reduce neuroinflammation, ameliorate metabolic dysfunction, and, perhaps most importantly, shift cerebral energy metabolism toward the utilization of ketone bodies instead of glucose, thereby addressing the energy deficit seen in the neurodegenerative brain. Evidence from studies on intermediate markers, such as improvements in cognitive scores like MoCA and reductions in plasma p-tau181, further supports this potential. Therefore, while it is premature to conclude that SGLT2 inhibitors are definitively effective in preventing dementia, the convergent evidence from real-world data and mechanistic studies provides a compelling argument for their potential. The current state of knowledge justifies cautious optimism and strongly supports the urgent need for definitive confirmation through focused, long-term, and properly designed RCTs.
